# Preliminary Study of Iron Concentration in the Human Placenta in Twin Pregnancies

**DOI:** 10.3390/biom13050749

**Published:** 2023-04-26

**Authors:** Konrad Grzeszczak, Patrycja Kapczuk, Patrycja Kupnicka, Maciej Mularczyk, Sebastian Kwiatkowski, Dariusz Chlubek, Danuta Kosik-Bogacka

**Affiliations:** 1 Department of Biology and Medical Parasitology, Pomeranian Medical University in Szczecin, Powstańców Wielkopolskich 72, 70-111 Szczecin, Poland; 2 Department of Biochemistry and Medical Chemistry, Pomeranian Medical University in Szczecin, Powstańców Wielkopolskich 72, 70-111 Szczecin, Poland; 3 Chair and Department of Human and Clinical Anatomy, Pomeranian Medical University in Szczecin, Powstańców Wielkopolskich 72, 70-111 Szczecin, Poland; 4 Department of Obstetrics and Gynecology, Pomeranian Medical University in Szczecin, Powstańców Wielkopolskich 72, 70-111 Szczecin, Poland; 5 Independent Laboratory of Pharmaceutical Botany, Pomeranian Medical University in Szczecin, Powstańców Wielkopolskich 72, 70-111 Szczecin, Poland

**Keywords:** iron, multiple pregnancy, human placenta

## Abstract

Background: Pregnancy significantly increases the demand for iron (Fe) in the female body to facilitate maternal blood volume expansion, placental development, and fetal growth. As Fe flux in pregnancy is significantly influenced by the placenta, the aim of this study was to determine the dependencies between the Fe concentration in the placenta, the infant’s morphometric parameters and the woman’s morphological blood parameters in the last trimester. Methods: The study was conducted on 33 women with multiple (dichorionic–diamniotic) pregnancies from whom the placentas were drawn, and their 66 infants, including pairs of monozygotic (n = 23) and mixed-sex twins (n = 10). Fe concentrations were determined based on inductively coupled plasma atomic emission spectroscopy (ICP-OES) using ICAP 7400 Duo, Thermo Scientific. Results: The results of the analysis showed that lower placental Fe concentrations were associated with deteriorated morphometric parameters of infants, including weight and head circumference. Although we found no statistically significant dependencies between Fe concentration in the placenta and the women’s morphological blood parameters, higher Fe concentration in the placenta of mothers supplemented with Fe correlated with better morphometric parameters in infants compared to those whose mothers received no Fe supplementation. Conclusions: The research adds additional knowledge for placental iron-related processes during multiple pregnancies. However, many limitations of the study do not allow detailed conclusions to be assessed, and statistical data should be assessed conservatively.

## 1. Introduction

Pregnancy is a dynamic process associated with significant physiological changes that affect the metabolism of nutrients. These adaptations are largely mediated by the placenta, which is a temporary fetal organ that plays protective, metabolic, transport and endocrine system functions [1]. It protects the fetus against xenobiotics and infections [2], and it releases hormones into the mother’s blood circulation (e.g., human chorionic gonadotropin (hCG)) or to the fetus (e.g., human chorionic somatomammotropin (HSC)) [3].

The placenta has a major impact on iron (Fe) transportation to the fetus. The iron flow through the placenta is unidirectional, estimated at several milligrams a day [4,5,6]. Iron is an essential element for a healthy pregnancy, playing a significant role in many biochemical processes such as electron transfer reactions, gene regulation, oxygen transport and storage, and regulation of cell growth and differentiation [7]. In pregnancy, the overall demand for Fe increases dramatically as the mother’s blood volume expands and the fetus grows and develops. It is estimated that the demand for Fe during pregnancy amounts to 740 mg of Fe during the entire pregnancy (the net pregnancy Fe loss to the mother), which must be provided through an appropriate diet and supplements [4]. Moreover, it is advised to accumulate Fe before pregnancy—the quantity of additional Fe should amount to 300 mg [8].

The increased demand for Fe in pregnancy is associated with the increased demands of the fetoplacental unit, to expand maternal erythrocyte mass and compensate for Fe loss at delivery [9]. The demand for Fe increases gradually until the third trimester. It ranges from 1 mg/day in the first trimester to 7.5 mg/day in the third [10]. In most cases, the woman’s body is not able to compensate for the loss of Fe during a singleton pregnancy; therefore, a preventive supplementation from 30 to 60 mg of Fe is applied [11]. For multiple pregnancies, this amount should probably be doubled. However, there is little research in this area.

**Figure 1:**
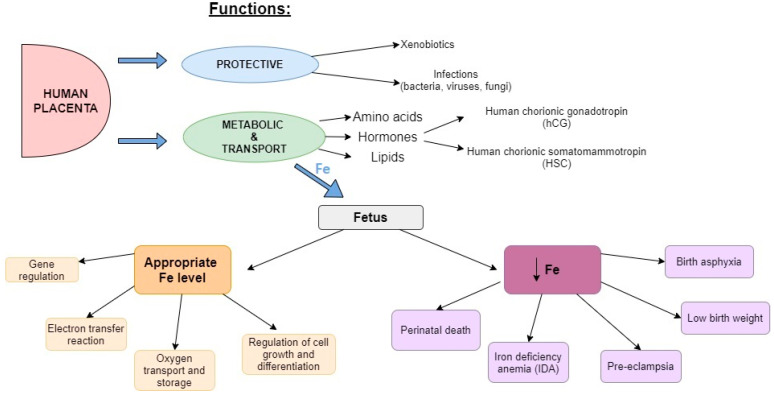
The placenta has several functions, of which the most important are protective, metabolic and transport functions. The protective function is crucial during infections (bacteria, viruses, fungi) or the action of xenobiotics. Transport and metabolic functions require many elements (e.g., Fe) and chemical compounds (e.g., amino acids, hormones or lipids). Appropriate levels of placental Fe are crucial for correct gene regulation, electron transfer reaction, oxygen transport and storage, and the regulation of cell growth and differentiation. Deficiency of placental Fe causes perinatal death, IDA, birth asphyxia, low birth weight, and pre-eclampsia.

A multiple pregnancy entails many complications which may occur due to Fe deficiency. The deficiency of Fe adversely affects fetal organogenesis and leads to birth asphyxia [12]. It may result in low birth weight, pre-eclampsia and iron deficiency anemia (IDA) in the mother [9] (Figure 1). Women with multiple pregnancies are at higher risk of antenatal and intrapartum complications [13], and they are associated with higher mortality rates [14]. Maternal physiological adaptation in twin pregnancies is exaggerated, and the rate of almost all maternal and fetal complications in twin pregnancies is higher than in singleton pregnancies [15]. Therefore, it can be considered that the parameters associated with twin pregnancies are not unique compared to singleton pregnancies but more intensified. A mechanism of Fe balance in the pregnant woman’s body, in both those with a single and multiple pregnancy, has been meticulously described in Grzeszczak et al. [6]. In spite of proper education and access to a balanced diet, 38% of women in the world develop IDA [16]. Unfortunately, in developing countries, the women’s perinatal death rate due to iron deficiency amounts to 20% of all perinatal deaths [17].

The aim of this study was to assess the dependencies between the Fe concentration in the placenta and the infant’s morphometric parameters and women’s morphological parameters in the last trimester.

## 2. Materials and Methods

The study was approved by the Biometric Committee of the Pomeranian Medical University in Szczecin (KB-0012/76/14 from 12 October 2014) and was carried out in accordance with the Declaration of Helsinki. The patients who participated in the study were notified about the study’s process and signed an agreement beforehand. At any stage of this project, the participants had the option to resign from it.

Placentas were collected from 33 pregnant Caucasian women from Poland (*n* = 32) and Ukraine (*n* = 1) admitted to the Obstetrics and Gynecology Clinic of the Independent Public Clinical Hospital No. 2 of the Pomeranian Medical University in Szczecin, Poland, at parturition between February 2015 and January 2021. Twin pregnancies were conceived naturally. The criterion for inclusion was multiple pregnancy, while the exclusion criteria were pre-eclampsia and fetal genetic anomaly. According to these criteria, we selected 66 newborns, including 23 pairs of monozygotic and 10 pairs of mixed-sex twins (females *n* = 31; males *n* = 35), and their mothers (*n* = 33). Birth weight, length and head circumference were registered at the time of birth using standard anthropometric procedures. Gestational age was calculated based on the first day of the last menstrual period according to Naegele’s rule and determined by the crown–rump length (CRL) measured during ultrasonography (USG) in the first trimester. The anthropometric and biological characteristics of mothers (age, weight, morphological blood analysis) and infants (shoulder width, weight, length, head circumference, gestational age and sex), and the weight and length of the umbilical cord, were collected from medical records. Each subject had to fill in a questionnaire on lifestyle behaviors (cigarette smoking, use of dietary supplements, etc.) (Table 1 and Table 2).

Placentas were collected immediately after delivery and then weighed and measured. A representative 10–15 cm long sample was excised from the middle of the radius (distance between the insertion of the umbilical cord and the periphery) without fetal and maternal membranes.

Afterbirth tissues were stored at −27 °C until the study group was gathered. Before the analysis, the samples were restored to room temperature, dried for three weeks at 55 °C and later at 105 °C for seven days to a constant weight. The prepared material was ground to a powder in a porcelain mortar to facilitate the digestion process [18].

Then, 0.2 g of each sample was placed in a clean polypropylene tubes. Afterwards, 5 mL of 65% HNO_3_ (Suprapur, Merck) and 1 mL of non-stabilized 30% H_2_O_2_ solution (Suprapur, Merck) were added to the samples to start the digestion process. HNO_3_ is a strong oxidizing acid that forms soluble salts with metals, which makes it appropriate for elemental analysis. The NO_2_ released during the digestion process in the presence of hydrogen peroxide forms HNO_3_, allowing the reaction to continue [19].

The reagents were added to 66 vials, and each sample was allowed 30 min pre-reaction time in a clean room, according to the manufacturer’s instructions, to discharge the excess of gas prior to sealing the vessels. Once the addition of all reagents was complete, the 66 samples were placed in Teflon vessels and heated (up to 180 °C) in the microwave digestion system MARS 5, CEM. The system allows the destruction of organic matter and with the use of concentrated nitric acid employed, carbohydrates matrices are rapidly decomposed at 140 °C, proteins at 150 °C, and lipids at 160 °C [19]. The closed-vessel system increases the efficiency of the digestion and is very often used in sample preparation for inorganic analysis using spectrochemical techniques [20,21], and nitric oxides formed during the process are continuously reabsorbed into the solution, allowing HNO_3_ to be formed until no more oxygen is available in the gas phase [19].

The samples were then transferred to acid-washed 15 mL polypropylene tubes. Iron levels were determined using inductively coupled plasma atomic emission spectroscopy (ICP-OES) by means of ICAP 7400 Duo, Thermo Scientific (Waltham, MA, USA). Analysis was performed in axial mode. Final 25-fold dilutions were performed prior to ICP-OES measurement. Blank samples were prepared by adding concentrated nitric acid to tubes without a sample and subsequently diluted in the same manner as the test samples. Multi-element calibration standards (ICP multi-element standard solution IV, Merck, Germany) were prepared with different concentrations of inorganic elements in the same manner as in blanks and samples. Deionized water (Direct Q UV, Millipore, approximately 18.0 MΩ) was used for the preparation of all solutions. Validation was performed by evaluating the NIST SRM 8414 reference material (National Institute of Standards and Technology, USA; reference values (mg/L) 71.2 ± 9.2; percentage of reference values 75.8) and the recovery of internal standard (yttrium; Y). The results were corrected by the software (Qtegra, Thermo Scientific) based on the recovery of Y, which was within 89–105% [22]. The r_2_ values for all standard curves ranged between 0.998 and 1.000. To eliminate possible interference, the emission lines were selected empirically in pilot measurement. The wavelength (nm) was 238.204.

The characteristics of mothers and their newborns are shown in Table 1 and Table 2. All infants were born without anemia. Most women (*n* = 26) took the supplement Prenatal DUO containing iron (II) fumarate 30 mg, which was consumed once daily from the beginning of pregnancy, and supplement intake was assessed during the medical interview at periodic meetings. Information on maternal diet during pregnancy was not available. Moreover, some women smoked cigarettes before pregnancy (*n* = 6; 2–10/per day).

Statistical analysis was performed using Statistica v13.0 (Stat Soft). Shapiro–Wilk analysis was performed to examine the normality of the data distribution. Due to the non-normal distribution of data, the Spearman rank correlation was used to analyze the relationship between the variables, determining the value of the coefficient and the level of statistical significance (rho, ρ). The Mann–Whitney test, which is the recommended non-parametric equivalent of the Student’s *t*-test, was used to compare the independent variables (two groups). This test can also be used for small group sizes (<20), in which case its U value should be given, while for large groups (>20), its Z value should be given. The Table 3 shows the Z value of the test, which was replaced with the U value for variables with a size of <20. The significance level of the analyses performed was assumed for the value of *p* < 0.05.

## 3. Results

The average water content in the placenta was approximately 83%. The concentrations of Fe in the placenta are presented in Table 4. Fe concentration in the placenta from the women ranged from 189.2 to 747.7 mg/kg dry mass (dw) (Table 4).

In the study, we determined correlations between Fe concentration in the placenta and the parameters of the infants, maternal characteristics, gestational age and maternal morphology (Table 5). We found a correlation in placenta Fe concentration and infant’s weight (ρ = −0.30) and the circumference of the infant’s head (ρ = −0.30).

In addition, we compared the obtained values of parameters defining maternal and child characteristics in relation to declared supplementation and smoking before pregnancy, normal and abnormal centiles of weight and length (Table 3).

We found significant higher values of placenta weight (*p* < 0.001), infant’s shoulder width (*p* < 0.001), infant’s weight (*p* < 0.01), and circumference of the infant’s head (*p* < 0.05) in the supplementation group compared to non-supplementing mothers. After accounting for cigarette smoking, significantly higher maternal MCV (*p* < 0.001), maternal MCH (*p* < 0.001) and maternal MCHC (*p* < 0.01) were found in the group of non-smoking mothers compared to smokers.

Moreover, children with normal centiles for length were born to mothers significantly older (gestational age, *p* < 0.05) and with a heavier placenta, *p* < 0.05). Similarly, children with normal percentiles for weight were born to significantly older mothers (gestational age, *p* < 0.05), had a heavier placenta (*p* < 0.05), and had a larger infant’s shoulder width (*p* < 0.01).

Finally, significance was tested between gender Fe concentrations. The average (standard deviation) iron for females (*n* = 31) was 476.5 (±105.7) and that of males (*n* = 34) was 416.1 (±119.4). Student’s *t*-test was used and found to be significantly different between the genders: t = 2.15 (*p* = 0.04).

In addition, Tukey’s RIR test was performed to compare the concentration of iron in the placentas of twins from the present study with the concentration of iron in placentas from single pregnancies in our previous research [18]. The test revealed a high level of statistical significance between the placental iron concentration in single and multiple pregnancies for both T1 Total (*p* = 0.00) and T2 Total (*p* = 0.00).

The scientific community has recently become interested in multiple pregnancies because of their significant increase in occurrence [23]. Since the 1980s, the global index of twin pregnancies has increased by 1/3, from 9.1 to 12 per 1000 births, which means 1.6 million twin pairs a year are born [24].

Iron is one of the most crucial elements for humans [25]. Neonatal Fe status is primarily a function of Fe transport from the mother to the fetus via the placenta in the third trimester [4]. In this study, we determined Fe concentration in placenta tissue collected from multiple-pregnant women (*n* = 33). Using Spearman’s correlation, we compared Fe concentrations with the anthropometric and clinical parameters of mothers and infants. In addition, we used relationships between selected lifestyle parameters of the examined women and infants’ morphometric parameters or women’s morphological parameters before the labor. In this study, Fe concentration in the placenta in multiple pregnancies was 444.9 mg/kg dw. The lower Fe concentration was observed in the placenta in women carrying multiples from the USA (17.94–34.16 mg/kg dw) [26]. In single pregnancy studies, Fe concentrations ranged from 252.16 to 1290.2 mg/kg [18,19,20,21,22,23,24,25,26,27]. In our previous research on Fe concentrations in placenta obtained from women with singleton pregnancies from the same study area, the Fe concentration in the placenta was found to average 640.73 mg/kg dw [15]. Depending on the study, the differences in mean Fe concentrations are probably associated with the different assay methods used. Mbofung et al. [27] and Reddy et al. [28] determined Fe levels using atomic absorption spectrometry (AAS), while in our study, we used ICP-OES. Differences in the economic status of the studied women and their access to health services in Nigeria [27], India [28], Nepal [29], Indonesia [30], and the USA [26,31], and the conditions prevalent at medical laboratories (e.g., differences in temperature or humidity), could have also affected this.

In the study presented, as well as in studies conducted by other authors, the focus was on analyzing the total iron content in placentas as a means of assessing their iron status. However, it is important to note that the placenta is a highly vascularized tissue, which means that the total iron content does not accurately reflect the amount of iron transferred to the fetus. To obtain a more accurate assessment, it would be necessary to investigate the concentration of both non-heme and heme iron. It is worth mentioning that Georgieff et al. [32] have concluded that the concentration of non-heme iron in the placenta is sufficient for assessing its iron status. However, two mechanisms may be responsible for the transport of heme iron to the fetus: endocytosis mediated by LDL-related protein 1 receptor (LRP1) and transmembrane transport with the use of ferroportin (FPN). LRP1 is highly expressed in the human placenta [33], and recent research suggests that it may also exist as a receptor for the hemopexin–heme complex. The authors suggest that a high expression of LRP1 in the human placenta could enable the delivery of heme iron to the fetus [34]. Similarly, FPN, which is under the strong control of hepcidin, is responsible for the export of inorganic iron released by heme oxygenase due to the lysosomal degradation of hemopexin. One of the tasks of hepcidin is to degrade the FPN protein and consequently limit cellular iron export [35]. Therefore, it must be considered that both placental LRP1 and placental FPN are responsible for the transfer of heme iron to the fetus.

Our study did not demonstrate any correlations between Fe concentration in the placenta and women’s blood morphological parameters. These results may be related to the small size of the study group of women and the fact they live in Poland, which is a country with relatively easy access to health services where doctors usually recommend appropriate supplementation to pregnant patients who can usually afford it. In contrast, women living in developing countries cannot balance the Fe deficiency with diet or supplementation by virtue of limited or no access to supplements, as well as inappropriate policies for preventing IDA in particular countries (e.g., India) [25]. In some countries, pregnant women are also exposed to additional complications—parasites such as hookworm, schistosomiasis, and malaria, as well as viruses (HIV) and bacteria (tuberculosis), which prevent women from maintaining proper Fe levels [36].

In the present study, the mean BMI and weight gain during pregnancy in the studied women were normal. This indicates that the diet of the studied women during pregnancy was adequate. Tan et al. [37] noted that underweight before pregnancy and rapid weight gain during pregnancy can lead to the development of IDA in women. Moreover, Jones et al. [38] found that obesity during pregnancy can lead to significant Fe deficiency. This may be due to a diet low in micronutrients, including Fe. In addition, obesity is accompanied by inflammation, which causes an inhibition of Fe absorption. Phillips et al. [39] have shown that obesity during pregnancy and excessive weight gain are independent risk factors. Tussing-Humphreys et al. [40] have shown that placental Fe transport does not differ between obese and non-obese women. In addition, in the present study, there was no correlation between a woman’s weight, BMI or weight gain during pregnancy and placental Fe concentration (Table 5). However, we showed a correlation between infant’s weight and maternal weight gain during pregnancy (Table 5). We thus confirmed the reports of Ludwig and Currie [41].

Scientific research has shown that Fe supplementation can restore sufficiency and resolve anemia [42]. Women expecting twins are more prone to IDA compared to single pregnancies [43]. A study by Shinar et al. [44] shows that in women with twin pregnancies and exhibiting IDA, doubling the daily dose of iron increases hemoglobin and ferritin. However, Ali et al. [45] demonstrated that a double dose of Fe does not have a significant influence on IDA prevention. Abbas et al. [46] performed a randomized controlled clinical trial on women in bigeminal pregnancy without anemia and showed that additional supplementation of Fe was not beneficial. The difference between these studies is the study group selection, as Shinar et al. [44] studied women who suffered from IDA, whereas Ali et al. [45] and Abbas et al. [46] selected women without IDA. Despite different approaches, the results in all studies are equally valuable, since Shinar et al. [44] proved that if IDA occurs, a double dose of Fe should be applied; however, if women can compensate for the iron deficit in pregnancy, then a single prophylactic dose of Fe is sufficient.

In our paper, a lower concentration of Fe worsened the morphometric parameters of infants, including weight, and head circumference (Table 5). This is in line with a study by Gambling et al. [47], where rats born to female rats with Fe deficiency were smaller than the control group. Gambling et al. [48] went on to scrutinize the influence of the lack of Fe supplementation on the offspring’s size during the rat’s pregnancy, and the results were wholly consistent with the previous study where the group of female pregnant rats which were supplemented with Fe gave birth to statistically bigger offspring than the control group.

This study shows the effect of a pregnant woman’s supplementation with Fe on the infant’s growth. The higher the Fe concentration in the placenta, the better the infant’s morphometric parameters were in comparison to those whose mothers were not supplemented with Fe (Table 3). Supplementation affects the morphometric levels of the newborn, as shown by Cogswell et al. [49]. They studied pregnant women (*n* = 513) in the USA who received Fe supplementation and reported that they birthed babies with significantly higher average birth weights and had a significantly lower incidence of low birth weight. This relationship was confirmed by Alwan et al. [50] in a prospective cohort study of pregnant women (*n* = 1274), where they showed an association between the intake of supplemental Fe and a higher birth weight. Peña-Rosas et al. [51] conducted a review of randomized or quasi-randomized trials by considering 61 trials including 44 studies involving 43,274 pregnant women. They showed that women supplemented with Fe during pregnancy were less likely to have children with low birth weight, although the authors emphasize that they are not sure of their results, as they did not reach statistical significance. Finally, Figueiredo et al. [52], after a systematic review and meta-analysis of 7243 articles, formulated a clear conclusion that anemia is a risk factor for low birth weight.

Smoking during pregnancy increases the risk of spontaneous abortions, ectopic pregnancies, fetal growth restriction, and prenatal placental abnormality [53]. The women surveyed in this study strongly denied any cigarette smoking during pregnancy, but they had regularly smoked before pregnancy or before they learned about the pregnancy. Our study showed a correlation between smoking before pregnancy and reduced levels of MCV, MCH and MCHC (indicating the deformation of erythrocytes), although we found no correlation with the level of hemoglobin (Table 3). These results are in line with the findings of Aldosari et al. [54], who report the adverse effect of smoking on erythrocyte shape in pregnant women who either smoked or were exposed to passive smoking. A lack of effect of smoking before pregnancy on hemoglobin levels in our study is interesting in the light of findings reported by Van Tiel et al. [55], who showed that an improvement in HgB and HCT in people who quit smoking takes as long as 2 years. This might indicate that Fe supplementation in pregnant women in our study accelerated the recovery of HgB and HCT levels over the period of pregnancy but was not sufficient to repair the erythrocyte structure (MCV, MCH, MCHC).

## 4. Limitations and Strengths

This work has certain limitations. We acknowledge that the studied women only represented a small part of pregnant women in northwestern Poland. Secondly, the placental transfer of Fe was not analyzed by the measurement of proteins most involved in iron transport into placental cells, including transferrin receptor (TfR) and ferroportin (FPN). The hormone responsible for Fe transport to the placenta, hepcidin, was not measured either due to the possible association of hepcidin and inflammation, which could consequently lead to IDA [56]. Thirdly, in the lack of similar articles, we had to include articles with infant blood (cord blood), in which iron was examined. A shortcoming of this study is the lack of analysis of the influence of external factors, including diet, environmental pollutants, the participant’s daily routine, and stress. Furthermore, we did not consider placental mass, position in the uterus, vascularity and function (determined by Doppler and biochemical studies such as PLGF), which can have a decisive influence on the physical development of the child. Nonetheless, our data on Fe concentration in the placenta and their comparison with morphometric parameters of the child significantly emphasize the importance of this element in the child’s development. The lack of correlation between a woman’s weight gain during the whole pregnancy vs. the concentration of Fe in the placenta indirectly confirms the latest data on this subject. Finally, this study shows an indirect but still significant effect of Fe supplementation on the placenta and fetus.

## 5. Conclusions

The research adds additional knowledge for placental iron-related processes during multiple pregnancies. However, many limitations of the study do not allow detailed conclusions to be assessed, and statistical data should be assessed conservatively.

## Figures and Tables

**Table 1:** Maternal and neonatal characteristics (AM, arithmetic mean; Med, median; SD, standard deviation; −/+95% CL, confidence interval; MCV, mean corpuscular volume; MCH, mean cell hemoglobin; MCHC, mean corpuscular hemoglobin concentration).

Parameter		AM ± SD	−95% CL	+95% CL	Med	Range
Maternal Characteristics						
age (years)		31.0 ± 4.2	3.55	5.02	30	21–41
BMI before pregnancy		23.5 ± 3.6	2.92	4.48	22.9	18.4–34.2
weight (kg) before pregnancy		65.5 ± 11.4	9.34	14.19	65	50–100
weight (kg) before delivery		83.9 ± 4.2	13.27	18.77	81.7	63–138
Weight gain during pregnancy		19.4 ± 8.6	7.08	10.76	16.0	8–38
red blood cell indices	HgB (mmol/L)	7.7 ± 0.9	0.76	1.08	7.5	5.7–9.7
	HCT (L/L)	0.361 ± 0.004	0.03	0.04	0.362	0.266–0.436
	MCV (fl)	87.7 ± 5.7	4.81	6.79	89.0	73.4–100.3
	MCH (fmol)	1.87 ± 0.16	0.14	0.19	1.86	1.46–2.28
	MCHC (mmol/L)	21.3 ± 0.7	0.59	0.84	21.3	19.9–22.7
Neonatal characteristics						
gestational age—weeks		35 ± 2	1.81	2.56	36	27–38
birth weight (g)		2376.6 ± 467.3	397.32	561.80	2485	690–3350
length (cm)		49.0 ± 4.0	3.37	4.77	50	29–55
head circumference (cm)		32.4 ± 2.1	1.76	2.49	33	25–37
shoulder width (cm)		29.8 ± 2.9	2.47	3.49	30	18–36
placenta weight (g)		519.7 ± 102.5	87.13	123.20	500	330–800
length of the umbilical cord (cm)		53.8 ± 8.2	6.92	9.79	54	28–75

**Table 2:** Smoothed centiles for birth weight and birth length of boys (*n* = 34) and girls (*n* = 31) (Fenton Growth Chart).

Centiles for Length (cm)	Boys	Girls	Total	Centiles for Birth Weight (kg)	Boys	Girls	Total
<3	2	0	2	<3	1	1	2
3–10	1	0	1	3	0	2	2
10–50	1	1	2	3–10	0	1	1
50	5	1	6	10	6	5	11
50–90	3	3	6	10–50	13	9	22
90	6	16	22	50	8	10	18
90–97	5	0	5	50–90	5	2	7
97	6	7	13	90	1	0	1
>97	6	3	9	97	0	1	1
				>97	1	0	1

**Table 3:** Mann–Whitney test results for intergroup comparison: the infants weight, supplementation and cigarette smoking before pregnancy.

	Supplementation	Med		Smoked Cigarettes before Pregnancy	Med		Centiles for Length	Med		Centiles for Birth Weight	Med	
		Supplementation	Non-Take Supplementation		Cigarette no Smoking #	Cigarette Smoking #		NormalCentiles for Length	AbnormalCentiles for Length		NormalCentiles for Birth Weight	AbnormalCentiles for Birth Weight
Gestational age							2.41 *	36	34	2.58 **	36	34
Placenta weight	−3.59 ***	520	435				2.32 *	510	430	2.07 *	500	430
Infant’s shoulder width	−3.24 ***	31	28							2.45 **	30	25
Infant’s weight	−2.62 **	2540	2055									
Circumference of the infant’s head	−2.31 *	33	31.5									
MCV maternal				2.95 ***	89.3	83.5						
MCH maternal				3.02 ***	1.920	1.745						
MCHC maternal				2.46 **	21.4	20.5						

*** < 0.001; ** < 0.01; * < 0.05; ^#^ smoked cigarettes before pregnancy.

**Table 4:** Concentration of iron (Fe) in the placenta (AM, arithmetic mean; Med, median; Min, minimum; Max, maximum, SD, standard deviation; −/+95% CL, confidence interval; T1, twin 1, T2, twin 2). Fe concentrations are presented in mg/kg dry weight (dw).

	AM ± SD	−95% CL	+95% CL	Med	Min	Max
pairs of monozygotic twins						
T1 (*n* = 23)	465.87 ± 106.21	82.14	150.32	484.24	206.28	665.82
T2 (*n* = 23)	435.45 ± 143.58	110.47	205.19	429.14	189.19	747.67
T1 + T2 (*n* = 46)	451.00 ± 125.36	103.78	158.36	480.31	189.19	747.67
pairs of mixed-sex twins						
T1 (*n* = 10)	442.10 ± 83.46	57.41	152.37	423.96	331.33	589.42
T2 (*n* = 10)	420.31 ± 106.76	73.43	194.90	415.27	297.88	653.27
T1 + T2 (*n* = 20)	431.21 ± 93.93	71.44	137.20	423.96	297.88	653.27
Total						
T1 (*n* = 33)	458.67 ± 99.18	79.76	131.19	468.34	206.28	665.82
T2 (*n* = 33)	430.72 ± 131.63	105.53	175.00	423.94	189.19	747.67
T1 + T2 (*n* = 66)	444.91 ± 116.23	99.12	140.54	445.85	189.19	747.67

**Table 5:** Spearman’s coefficients for correlations between the Fe concentration in the placenta and the infant’s morphometric parameters and women’s parameters.

	Fe Concentration of Placenta	Gestational Age	Infant’s Weight	Infant’s Length	Placenta Weight	Circumference of the Infant’s Head	Maternal Age	Weight before Delivery	Weight before Pregnancy	HgB	HCT	MCV
Neonatal characteristics												
Infant’s weight	−0.30 **	0.63 ***										
Infant’s length		0.64 ***	0.63 ***									
Placenta weight		0.43 ***	0.41 ***	0.33 **								
Circumference of the infant’s head	−0.30 **	0.44 ***	0.79 ***	0.45 ***	0.39 ***							
Infant’s shoulder width		0.69 ***	0.78 ***	0.55 ***	0.52 ***	0.65 ***						
Maternal characteristics												
Maternal age						−0.28 ***						
Weight before delivery						0.40 **			0.81 ***			
Weight gain during pregnancy			0.36 **					0.54 ***				
BMI								0.66 ***	0.90 ***			
Weight before pregnancy												
Maternal morphology												
HgB							0.26 *					
HCT							0.26 *			0.94 ***		
MCV										0.26 *		
MCH										0.36 ***		0.91 ***
MCHC										0.56 ***	0.29 ***	

*** < 0.001; ** < 0.01; * <0.05.

## Data Availability

The data presented in this study are available on request from the corresponding author.
